# The effects of Gouqi extracts on Morris maze learning in the APP/PS1 double transgenic mouse model of Alzheimer’s disease

**DOI:** 10.3892/etm.2013.1006

**Published:** 2013-03-14

**Authors:** QIANLIN ZHANG, XIAOPING DU, YUPING XU, LEI DANG, LI XIANG, JIEWEN ZHANG

**Affiliations:** 1 Department of Neurology, Renmin’s Hospital, Zhengzhou, Henan 450006;; 2 Department of Neurology, Xiangya Hospital, Changsha, Hunan 450008, P.R. China

**Keywords:** Gouqi, Alzheimer’s disease, learning, memory, Morris maze

## Abstract

The present study examined the effects of Gouqi (*Lycium barbarum*) on the learning and memory abilities of an APP/PS1 double transgenic mouse model of Alzheimer’s disease. We employed a Morris water maze to examine the spatial memory in this mice line with or without Gouqi extracts treatment. We identified that 2 weeks of oral administration of Gouqi extracts at 10 mg/kg improved the performance of the APP/PS1 mice in the learning and the memory retrieval phases of the Morris maze. In correlation with this, the levels of Aβ(1–42) in hippocampal tissue were reduced by the Gouqi treatment. We conclude that pharmacological treatment with Gouqi extracts is beneficial at the later stages of Alzheimer’s disease.

## Introduction

Traditional herbal medicine is regularly used in clinical and pre-clinical studies of neurodegenerative diseases. One interesting herbal plant is Gouqi (*Lycium barbarum*), which has been widely used for the promotion of longevity and anti-aging in traditional medicine (1). The main bioactive components of Gouqi include polysaccharides, carotenoids and phytosterols (2,3). These molecules are of therapeutic potential in various types of disease. For instance, the polysaccharides are protective for neurons, as well as other organs being challenged, for example, during oxidative stress (4–6). Additionally, different forms of extracts from *Lycium barbarum* may reduce pathological inflammation (7). They also stimulate moderate immune responses and, therefore, may be used as vaccine adjuvants (8). Structure-based drug design has benefited from the polysaccharide extract of *Lycium barbarum*, leading to the development of new drugs (9).

However, the detailed effects of this plant, particularly the effective components, on Alzheimer’s disease (AD) have not been extensively investigated in animals undergoing behavioral tests (10–13). In the present study we administered the extracts to the APP/PS1 double transgenic mouse model of AD to investigate the potentially beneficial effects on learning and memory in the Morris maze. The results provide more evidence for the use of herbal medicines in the treatment of aging and neurodegenerative diseases.

## Materials and methods

### Animals

The APP/PS1 heterozygous mouse line [B6C3-Tg (APPswe, PSEN1de9) 85Dbo/J (005864)] was purchased from The Jackson Laboratory (Bar Harbor, ME, USA). The mice had free access to food and water, under a 12/12 h light/dark cycle. Genotyping was performed at 1–2 weeks after birth. A total of 30 male mice (20 double mutant mice, 10 normal mice) aged 12–13 months were included in the study. The mice were randomly assigned into three groups: a control group (10 normal mice treated with saline for 2 weeks), an APP/PS1 saline group (10 double mutant mice treated with saline for 2 weeks) and an APP/PS1 treated group (10 double mutant mice treated with Gouqi extracts at 10 mg/kg/day for 2 weeks).

The study was approved by the local ethics committee of animal research in Renmin’s Hospital (Zhengzhou, China).

### Drug

Gouqi extract was provided by Shenghe Pharmaco Co. (Henan, China) as a water soluble powder (>98% polysaccharides). Preliminary studies have shown it to be non-toxic to normal C57 mice at doses ≤500 mg/kg for 2 weeks, which is consistent with clinical observations.

### Morris maze behavior

The Morris water maze test was used to examine the changes in the learning and memory abilities of the mice, as previously described (14). In brief, a circular water maze was used. The diameter was 120 cm and the height was 50 cm. A hidden platform with a diameter of 9 cm was inside the maze and the surface was 0.5 cm below the surface of the water. Floating plastic particles were placed on the surface of the water to hide the platform from sight. The temperature of the water was 25.0±0.5°C. The experiment was performed in a double-blind manner. The individual who performed the experiment was familiar with the mice from the two weeks of oral feeding.

For the experiment, the mice were placed in a random area inside the maze for free swimming until they identified the hidden platform. The whole experiment lasted for 7 days. For the first 6 days, the mice were left in the maze to find the platform with a maximum time of 60 sec. The learning section was repeated 5 times each day, with an interval of 1 h between each session. On the last day, the platform was removed and the time that the mice spent in the memorized region was recorded over a period of 3 min (180 sec).

### Enzyme-linked immunosorbent assay (ELISA)

After the end of the behavioral tests, the animals were sacrificed and the brains were removed immediately and stored in ice-cold saline. The Aβ(1–42) content was measured with an Aβ(1–42) ELISA kit (R&D Systems, Minneapolis, MN, USA), following the manufacturer’s instructions. Briefly, bilateral hippocampi were isolated and homogenized in Tris-buffered saline (TBS) with protease inhibitors and ethylenediamine tetraacetic acid (EDTA), followed by sodium dodecyl sulfate (SDS) and Triton X-100 treatment, prior to centrifuge processing. Finally the soluble fraction was collected for ELISA analyses. Briefly, a double-antibody sandwich ELISA was performed. The optical density (OD) was measured at 490 nm in the plate reader. The OD values from the samples were calculated into the concentration based on the standard curve.

### Statistical analysis

The data are presented as mean ± standard deviation and statistical analysis was performed with SPSS 11.0 software (SPSS Inc., Chicago, IL, USA). Paired samples t-test and one-way analysis of variance (ANOVA) were used to determine the differences between groups. P<0.05 was considered to indicate a statistically significant difference.

## Results

### Treatment with Gouqi extracts improves learning ability in APP/PS1 mice

In the first 6 days we identified that the APP/PS1 mutant mice demonstrated impaired ability in water maze learning, compared with the control group (P<0.05); while the APP/PS1 mice pre-treated with Gouqi extracts for 2 weeks demonstrated improved learning ability on the first day of learning (P<0.05; Table I). Preliminary studies indicated no differences in locomotive behavior following Gouqi extract feeding for 2–4 weeks (unpublished data), suggesting that the decreased time following drug treatment is not due to an increased swimming speed. Therefore, these results suggest that Gouqi extracts improve the learning ability of mice.

### Treatment with Gouqi extracts enhances memory in APP/PS1 mice

On day 7, the control mice demonstrated enhanced searching behavior in the memorized region where the platform was (65.9±4.72% of time). However the APP/PS1 untreated mice spent significantly less time in the memorized region (32.12±7.41%, P<0.05), suggesting a loss of memory ability at this age. However, the treated group demonstrated enhanced memory ability (51.79±4.58%, P<0.05; Table II).

### Treatment with Gouqi extracts reduces Aβ(1–42) content in hippocampal tissue

The ELISA results indicate a reduction in Aβ(1–42) content in the hippocampal tissue following Gouqi treatment (Table III). In the control mice, the hippocampus contained an extremely small amount of Aβ(1–42), the level of which was high in the APP/PS1 mutant mice. However, upon treatment, the Aβ(1–42) level was reduced, suggesting that Aβ(1–42) is a possible target in the pharmacology of Gouqi extract.

## Discussion

The present study examined the effects of Gouqi extracts on the learning and memory ability in a transgenic mouse model of AD. The results demonstrated that pharmacological treatment with Gouqi extracts improves the learning behavior and stabilizes the memory in these aged mice with genetic defects. The mechanism may involve the downregulation of Aβ(1–42) accumulation in the brain.

The APP/PS1 mouse line is widely accepted as a mouse model for AD. The aged animal demonstrates significant loss of learning and memory behavior, as well as synaptic changes in a number of brain areas, including the hippocampus, cortex and cerebellum (15–17). Additionally, there are findings suggesting changes in neurovascular functions, as well as neuroinflammatory changes associated with the behavioral alterations (17,18).

There are several possible underlying causes for this improved learning behavior. It has previously been identified that Gouqi extracts have an anti-inflammatory effect in the neural system and downregulate the circulating pro-inflammatory molecules (1,4,7,19). In the mouse model of AD, neuroinflammation is considered a potential factor contributing to the memory loss and circuit dysfunction. Therefore, the downregulation of inflammatory signaling inside the brain may explain the beneficial effects that were observed. The other possibility is the direct regulation of synaptic transmission and neural circuit functions (1). Aβ(1–42) directly reduces synaptic transmission, particularly in hippocampal synapses. In the current study, we identified that Gouqi treatment decreases Aβ(1–42) content in the hippocampus. This suggests decreased plaque formation and better-preserved neural circuits. Gouqi extracts contain a number of different molecules and we are yet to isolate bio-active components for future studies.

From the perspective of AD prevention, Gouqi extracts have neuroprotective effects on numerous types of neurons (11,20). The administration of Gouqi extracts at the early phase of AD or in the risk stage, including in mild cognitive dysfunction, may be helpful. We expect to conduct further studies using younger mice.

## Figures and Tables

**Table I:** Improved learning ability in APP/PS1 mice treated with Gouqi extracts (time taken to find the hidden platform, sec).

Group	Day 1	Day 2	Day 3	Day 4	Day 5	Day 6
Control	46.16±3.28	42.19±4.09	37.33±2.1	30.27±4.02	21.09±5.52	16.31±4.92
APP/PS1 untreated	56.2±2.94^a^	54.33±9.91^a^	50.27±4.32^a^	42.72±6.73^a^	38.94±5.15^a^	38.22±6.94^a^
APP/PS1 treated	50.0±4.43^b^	47.34±5.02^b^	40.14±4.11^b^	34.79±5.45^b^	23.02±7.8^b^	20.2±5.66^b^

a P<0.05, compared with the control group;

b P<0.05, compared with the APP/PS1 untreated group.

**Table II:** Enhanced memory in APP/PS1 mice treated with Gouqi extracts (% of time spent in the memorized region).

Group	Day 7
Control	65.9±4.72
APP/PS1 untreated	32.12±7.41^a^
APP/PS1 treated	51.79±4.58^b^

a P<0.05, compared with the control group;

b P<0.05, compared with the APP/PS1 untreated group.

**Table III:** Reduced Aβ(1–42) content in hippocampal tissue (ng/*μ*g).

Group	Day 7
Control	3.1±2.42
APP/PS1 untreated	38.62±11.84^a^
APP/PS1 treated	17.2±9.36^b^

a P<0.05, compared with the control group;

b P<0.05, compared with the APP/PS1 untreated group.
